# *ABCB1* C3435T Polymorphism and Response to Clopidogrel Treatment in Coronary Artery Disease (CAD) Patients: A Meta-Analysis

**DOI:** 10.1371/journal.pone.0046366

**Published:** 2012-10-09

**Authors:** Jia Su, Jin Xu, Xiaojing Li, Han Zhang, Juwei Hu, Renyuan Fang, Xiaomin Chen

**Affiliations:** 1 Department of Cardiology, The Affiliated Ningbo No.1 Hospital, School of Medicine, Ningbo University, Ningbo, Zhejiang Province, People’s Republic of China; 2 Institute of Preventative Medicine, School of Medicine, Ningbo University, Ningbo, Zhejiang Province, People’s Republic of China; 3 Department of Biochemistry and Molecular Biology, School of Medicine, Ningbo University, Ningbo, Zhejiang Province, People’s Republic of China; Tor Vergata University of Rome, Italy

## Abstract

**Background:**

A number of investigators have evaluated the association between the *ABCB1* polymorphism and clopidogrel responding, but the results have been inconclusive. To examine the risk of high platelet activity and poor clinical outcomes associated with the *ABCB1* C3435T polymorphism in CAD patients on clopidogrel, all available studies were included in the present meta-analysis.

**Methods:**

We performed a systematic search of PubMed, Scopus and the Cochrane library database for eligible studies. Articles meeting the inclusion criteria were comprehensively reviewed, and the available data were accumulated by the meta-analysis.

**Results:**

It was demonstrated that the ABCB1 C3435T variation was associated with the risk of early major adverse cardiovascular events (MACE) (T vs. C OR, 1.34; 95% CI, 1.10 to 1.62; *P = *0.003; TT vs. CC: OR, 1.77; 95% CI, 1.19 to 2.63; *P* = 0.005; CT + TT vs.CC: OR, 1.48; 95% CI, 1.06 to 2.06; *P* = 0.02) and the polymorphism was also associated with the risk of the long-term MACE in patients on clopidogrel LD 300 mg (T vs. C: OR, 1.28; 95% CI, 1.10 to 1.48; *P* = 0.001; TT vs. CC: OR, 1.59; 95% CI, 1.19 to 2.13; *P* = 0.002; CT + TT vs.CC: OR, 1.39; 95% CI, 1.08 to 1.79; *P* = 0.01). The comparison of TT vs. CC was associated with a reduction in the outcome of bleeding (TT vs. CC: OR, 0.51; 95% CI, 0.40 to 0.66; *P*<0.00001). However, the association between *ABCB1* C3435T polymorphism and platelet activity and other risk of poor clinical outcomes was not significant.

**Conclusions:**

The evidence from our meta-analysis indicated that the *ABCB1* C3435T polymorphism might be a risk factor for the MACE in patients on clopidogrel LD 300 mg, and that TT homozygotes decreased the outcome of bleeding compared with CC homozygotes.

## Introduction

Clopidogrel inhibits the adenosine-diphosphate-induced platelet aggregation, reducing the cardiovascular complications in patients with coronary atherosclerotic heart disease (CAD), especially in those undergoing percutaneous coronary intervention (PCI) [1]. However, the pharmacodynamic response to clopidogrel varies greatly among patients [2], and patients with lesser degrees of platelet inhibition are more likely to experience recurrent ischemic events [3], [4]. Although the mechanisms have not been fully clarified, genetic polymorphisms may play a vital role in individual susceptibility to drug response [5].

The *ABCB1* (ATP-binding cassette, sub-family B, member 1, also called *MDR1* or *TAP1*) gene encodes the intestinal efflux transporter P-glycoprotein, which modulates the absorption of clopidogrel [6]. The *ABCB1* gene locates at 7, p21–21.1 [7], and more than 50 single-nucleotide polymorphisms (SNP) within this gene have been described in the literature. Among them, the *ABCB1* C3435T (rs1045642) is extensively studied and some research has been shown that the *ABCB1* C3435T genotype influences the impaired function of P-glycoprotein which can hinder the absorption of clopidogrel [8].

Antiplatelet response could be investigated through poor clinical outcomes and impaired response to antiplatelet therapy in the laboratory test. Simon *et al.* [9] first analyzed the effect of C3435T polymorphism on clinical outcomes in patients receiving clopidogrel and found that patients with TT genotype had a higher rate of subsequent cardiovascular events than those with CC genotype. One study [10] indicated that the *ABCB1* C3435T polymorphism influenced ADP dependent platelet reactivity and showed that T-allele carriers were likely to have a poor response to antiplatelet therapy in the lab test. However, the results from different studies [9]–[20] were inconsistent. Thus, in the present study, a meta-analysis was performed to delineate the association between *ABCB1* C3435T polymorphism and platelet activity as well as the risk of poor clinical outcomes in patients treated with clopidogrel.

**Figure 1:**
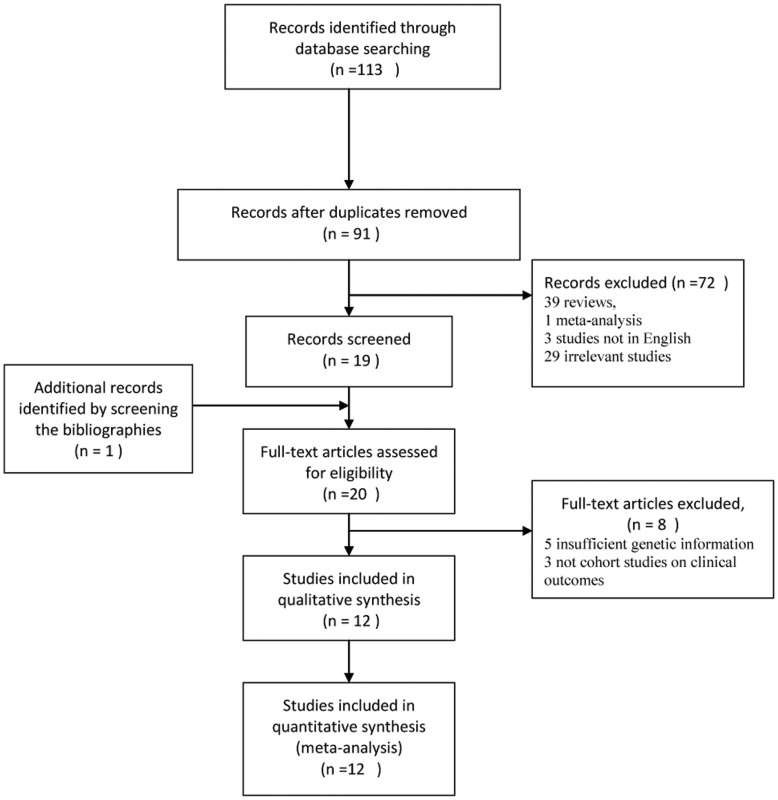
Flow diagram of the trial selection process.

**Table 1:** Main characteristics of studies included on platelet activity tests in the meta-analysis.

First author	year	ethnicity	population studied	treatment protocal	definition of case	case			control			HWE
						CC	CT	TT	CC	CT	TT	
Spiewak, M. [10]	2009	NA	ACS treated with PCI	LD aspirin 300 mg clopidogrel (300 mg or 600 mg)MD aspirin 75 mg clopidogrel 75 mg qd	collagen/adenosine diphosphate (CADP)-CT<130s	4	16	10	23	34	11	0.791
Kim, I. S. [12]	2012	Asian	Patients treated with PCI	cilostazol 100 mg bid, clopidogrel 75 mg and aspirin 200 mg qd	5 mol/l ADP-induced maximal PR (Aggmax)>46%.	7	4	1	45	58	12	0.287
Jeong, Y. H. [13]	2010	Asian	AMI treated with coron ary angiography or PCI	MD clopidogrel 150 mg aspirin 200 mg qd	5 mol/l ADP-induced maximal PR (PRmax)>50%.	13	14^a^		56^b^	55^b^	15^b^	0.791
Jeong, Y.H. [14]	2011	Asian	AMI treated with PCI	LD aspirin 300 mg clopidogrel 600 mgMD aspirin 100–200 mg clopidogrel 75 mg	20 mol/L ADP-induced maximal PR(PRmax)>59%	64	54	16	60	54	18	0.303

LD: loading dose; MD: maintenance dose; HWE: Hardy-Weinberg equilibrium.

a the number is consisted of CT and TT.

b the number is consisted of case group and control group.

**Table 2:** Main characteristics and genotype of studies included on the poor clinical outcomes in the meta-analysis.

First author	Year	ethnicity	Male gender,No. (%)	Hypertension, No. (%)	Diabetes,No. (%)	Hypercholester-olemia No. (%)	Previous or current smoker, No. (%)	Total			HWE
								CC	CT	TT	
Mega, J. L. [15]	2010	Caucasian(97.6)	1040(70.7)	1903(64.9)	321(21.8)	1424(48.6)	560(38.1)	330	727	414	0.750
Simon, T. [9]	2009	NA	1559(70.6)	1280(58.0)	698(31.6)	1088(49.3)	1206(54.6)	564	1050	574	0.060
Spiewak, M. [10]	2009	NA	69(70.4)	52 (53.1)	17(17.3)	35 (35.7)	43 (43.9)	26	44	18	0.938
Wallentin, L. [16]	2010	Caucasian (98)	3571 (69.0)	NA	1189 (23)	NA	3099(60.2)	1195	2518	1386	0.434
Tiroch, K. A. [17]	2010	NA	694(74.8)	691(74)	224(24.1)	482(52)	339(36.5)	203	457	268	0.755
Campo, G. [18]	2011	NA	231(77)	215 (72)	71(23.7)	153 (51)	71(23.7)	69	157	74	0.416
Delaney, J. T. [19]	2012	Caucasian	440(63.5)	560(80.8)	241(34.8)	643(92.8)	419(60.5)	173	336	179	0.543
Wang, X. D. [11]	2012	Asian	361(67.4)	305(56.9)	273(50.9)	295(55.0)	186(34.7)	364	161	11	0.478
Jeong, Y. H. [14]	2011	Asians	195 (73.3)	125 (47.0)	70 (26.3)	71 (26.7)	141 (53.0)	124	108	34	0.216
Jaitner, J. [20]	2012	Caucasian	1180(77.4)	1362(89.4)	430(28.2)	1068(70.1)	207(13.6)	444	740	340	0.342

NA, not applicable.

**Table 3:** Treatment characteristics of studies included on the poor clinical outcomes in the meta-analysis.

First author	Year	Population studied	Treatment protocal	Study period	The poor outcomes
Mega, J. L.	2010	ACS treated with PCI	LD clopidogrel 300 mg	15 months	stent thrombosis
			MD clopidogrel 75 mg qd		major or minor bleeding
					MACE (cardiovascular death, non-fatal myocardial infarction and non-fatal stroke)
Simon, T.	2009	AMI treated withcoronary angiography or PCI	LD clopidogrel 300 mg aspirin(98%)	12 months	outcome event (Death,nonfatal myocardial infarction or stroke)
Spiewak, M	2009	ACS treated with PCI	LD aspirin 300 mg clopidogrel(300 mg or 600 mg)	1.7 years	cardiovascular deaths and non-fatal myocardial infarction
			MD aspirin 75 mg clopidogrel75 mg qd		
Wallentin, L.	2010	Acute coronarysyndrome.	LD clopidogrel 300–600 mg,	12 months	Cardiovascular death, myocardial infarction, and stroke,
			MD clopidogrel 75 mg qd aspirin (96%)		Definite stent thrombosis
					Major bleeding
Tiroch, K. A.	2010	AMI treated withcoronary angiography	LD clopidogrel 600 mg	12 months	MACE(including death, MI, TLR, and stroke)
			MD aspirin 100 mg bid clopidogrel 75 mg qd		Stent thrombosis
Campo, G.	2011	Ischemic heart disease underwent PCI	LD aspirin 300 mg clopidogrel 600 mg	12 months	Ischemic adverse events(Death, MI, stroke, stent thrombosis)
			MD aspirin 300 mg clopidogrel75 mg qd		minor or major bleedings
Delaney, J. T.	2012	MI or treated with PCI	Clopidogrel not applicable	12–24 months	Primary endpoint cardiovascular events(all-cause mortality, MI, stroke, revascularization, and stent thrombosis)
Wang, X. D.	2012	Patients treatedwith PCI	LD aspirin 100 mg clopidogrel 300 mg	1 month	Major or Minor bleeding
			MD aspirin 100 mg clopidogrel75 mg qd		Early definite stent thrombosis
					MACE(included cardiovascular death, stent thrombosis, recurrent acute coronary syndrome)
Jeong, Y. H.	2011	AMI treated withcoronary angiography or PCI	LD aspirin 300 mg clopidogrel 600 mg	12 months	major or minor bleeding
			MD aspirin 100–200 mg clopidogrel 75 mg qd		MACE (cardiovascular death, nonfatal myocardial infarction, and ischemic stroke)
Jaitner, J.	2012	Patients treatedwith PCI	LD aspirin 500 mg clopidogrel 600 mg	14 months	stent thrombosis
			MD Aspirin 100 mg bid, clopidogrel 75 mg bid*3d then 75 mg qd		

LD: loading dose; MD: maintenance dose; HWE: Hardy-Weinberg equilibrium.

**Table 4:** The distribution of ABCB1 C3435T genotypes for patients with and without long-term MACE.

	T		C		TT		CC		TT		CT+CC		TT+CT		CC	
first author	event	total	event	total	event	total	event	total	event	total	event	total	event	total	event	total
Wallentin 2010 [16]	507	5290	509	4908	137	1386	138	1195	137	1386	371	3713	370	3904	138	1195
Simon 2009 [9]	318	2198	262	2178	85	574	57	564	85	574	205	1614	233	1624	57	564
Mega 2010 [15]	158	1555	106	1387	52	414	26	330	52	414	80	1057	106	1141	26	330
Campo 2011 [18]	28	305	14	226	8	74	1	69	8	74	13	226	20	231	1	69
Tiroch 2010 [17]	NA	NA	NA	NA	NA	NA	NA	NA	NA	NA	NA	NA	22	725	63	203
Spiewak 2009 [10]	12	80	8	96	3	18	1	26	3	18	7	70	9	62	1	26
Jeong 2011 [14]	7	176	19	356	1	34	7	124	1	34	12	232	6	142	7	124

**Table 5:** The distribution of ABCB1 C3435T genotypes for patients with and without early MACE.

first author	T		C		TT		CC		TT		CT+CC		TT+CT		CC	
	event	Total	event	total	event	total	event	total	event	total	event	total	event	total	event	total
Simon 2009 [9]	160	2198	128	2178	41	574	25	564	574	41	1614	103	1624	119	564	25
Mega 2010 [15]	102	1555	62	1387	35	414	15	330	414	35	1057	47	1141	67	330	15
Wang 2012 [11]	NA	NA	NA	NA	NA	NA	NA	NA	NA	NA	NA	NA	172	5	364	15

**Table 6:** The distribution of ABCB1 C3435T genotypes for patients with and without MI.

first author	T		C		TT		CC		TT		CT+CC		TT+CT				CC		
	event	total	event	total	event	total	event	total	event	total	event	total	event			total	event	total	
Mega 2010 [15]	NA	NA	NA	NA	NA	NA	NA	NA	48	414	70	1057	NA			NA	NA	NA	
Campo 2011 [18]	18	305	8	295	5	74	0	69	5	74	8	226	13			231	0	69	
Tiroch 2010 [17]	NA	NA	NA	NA	NA	NA	NA	NA	NA	NA	NA	NA	16			725	6	203	
Wang 2012 [11]	NA	NA	NA	NA	NA	NA	NA	NA	NA	NA	NA	NA	2			172	7	364	
Delaney 2012 [19]	33	694	43	682	6	179	11	173	6	179	32	509		27	515		11	173	

**Table 7:** The distribution of ABCB1 C3435T genotypes for patients with and without stroke.

first author	T		C		TT		CC		TT		CT+CC		TT+CT		CC	
	event	total	event	total	event	total	event	total	event	total	event	total	event	total	event	total
Wallentin 2010 [16]	41	5290	35	4908	13	1386	10	1195	13	1386	25	3713	28	3904	10	1195
Mega 2010 [15]	NA	NA	NA	NA	NA	NA	NA	NA	2	414	3	1057	NA	NA	NA	NA
Campo 2011 [18]	2	305	2	295	1	74	1	69	1	74	1	226	1	231	1	69
Tiroch 2010 [17]	NA	NA	NA	NA	NA	NA	NA	NA	NA	NA	NA	NA	7	725	1	203
Delaney 2012 [19]	1	694	1	682	NA	NA	NA	NA	0	179	1	509	1	515	0	173

**Table 8:** The distribution of ABCB1 C3435T genotypes for patients with and without mortality.

first author	T		C		TT		CC		TT		CT+CC		CT+TT		CC	
	event	total	event	total	event	total	event	total	event	total	event	total	event	total	event	total
Mega 2010 [15]	NA	NA	NA	NA	NA	NA	NA	NA	5	414	8	1057	NA	NA	NA	NA
Campo 2011 [18]	8	305	4	295	2	74	0	69	2	74	4	226	6	221	0	69
Tiroch 2010 [17]	NA	NA	NA	NA	NA	NA	NA	NA	NA	NA	NA	NA	47	725	17	203
Delaney 2012 [19]	12	694	16	682	4	179	6	173	4	179	10	509	8	515	6	173

**Table 9:** The distribution of ABCB1 C3435T genotypes for patients with and without thrombosis.

first author	T		C		TT		CC		TT		CT+CC		TT+CT		CC	
	event	total	event	total	event	total	event	total	event	total	event	total	event	total	event	total
Wallentin2010 [16]	56	3487	62	3239	14	917	17	793	14	917	45	2446	42	2570	17	793
Mega 2010 [15]	NA	NA	NA	NA	NA	NA	NA	NA	5	392	12	1004	NA	NA	NA	NA
Campo 2011 [18]	6	305	2	295	2	74	0	69	2	74	2	226	4	231	0	69
Tiroch 2010 [17]	NA	NA	NA	NA	NA	NA	NA	NA	NA	NA	NA	NA	7	725	3	203
Wang 2012 [11]	NA	NA	NA	NA	NA	NA	NA	NA	NA	NA	NA	NA	1	172	5	364
Delaney 2012 [19]	7	694	15	682	1	179	5	173	1	179	10	509	6	515	5	173
Jaitner 2012 [20]	69	1420	63	1628	19	340	16	444	19	340	47	1184	50	1080	16	444

**Table 10:** The distribution of ABCB1 C3435T genotypes for patients with and without bleeding.

first author	T		C		TT		CC		TT		CT+CC		TT+CT		CC	
	event	total	event	total	event	total	event	total	event	total	event	total	event	total	event	total
Wallentin 2010 [16]	519	5272	477	4884	137	2508	116	1188	137	1382	361	3696	382	3890	116	1188
Mega 2010 [15]	NA	NA	NA	NA	NA	NA	NA	NA	15	414	26	1052	NA	NA	NA	NA
Campo 2011 [18]	16	305	22	335	4	157	7	69	4	74	15	226	12	231	7	69
Jeong 2011 [14]	5	176	11	356	1	108	4	124	1	34	7	232	4	142	4	124
Wang 2012 [11]	NA	NA	NA	NA	NA	NA	NA	NA	NA	NA	NA	NA	10	172	20	364

NA, not applicable.

## Methods

### 1. Literature Search

Three electronic databases (PubMed, Scopus and the Cochrane library) were searched (the last search was updated in March 2012 with the following terms combined: antiplatelet, clopidogrel, aspirin, platelet activity, *ABCB1*, *MDR1*, multidrug resistance, polymorphism). All eligible studies were retrieved and their bibliographies as well as the previous meta-analysis were checked for other relevant studies.

**Figure 2:**
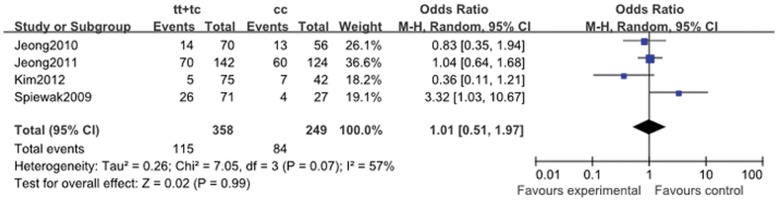
Pooled random-effects-based odds ratio of platelet activity associated with *ABCB1* C3435T polymorphism. Comparison: TT+TC vs. CC.

**Table 11:** The total and stratified analysis of the ABCB1 C3435T polymorphism on antiplatelet responding.

	T vs. C			TT vs. CC			TT vs. CC + CT			CT + TT vs.CC		
Variables	OR(95%CI)	*P* a	*P*	OR(95%CI)	*P* a	*P*	OR(95%CI)	*P* a	*P*	OR(95%CI)	*P* a	*P*
Platelet activity	1.06 (0.53, 2.13)	0.02	0.86	1.36 (0.35, 5.30)	0.05	0.66	1.20 (0.69, 2.08)	0.19	0.53	1.01 (0.51, 1.97)	0.07	0.99
MACE	1.16 (0.94, 1.45)	0.01	0.17	1.39 (0.86, 2.24)	0.007	0.18	1.26 (0.98, 1.63)	0.01	0.08	1.09 (0.77, 1.54)	0.008	0.62
LD 600 mg	1.13 (0.55, 2.29)	0.19	0.74	2.05 (0.13, 31.97)	0.07	0.61	1.48 (0.51, 4.29)	0.27	0.47	1.06 (0.43, 2.64)	0.12	0.09
LD 300 mg	1.28 (1.10, 1.48)	0.53	0.001	1.59 (1.19, 2.13)	0.79	0.002	1.42 (0.98, 2.06)	0.01	0.07	1.39 (1.08, 1.79)	0.43	0.01
others	1.09 (0.61, 1.93)	0.16	0.78	1.24 (0.32, 4.88)	0.17	0.76	1.00 (0.81, 1.22)	0.48	0.99	1.20 (0.32, 4.56)	0.15	0.79
MACE early	1.34 (1.10, 1.62)	0.39	0.003	1.77 (1.19, 2.63)	0.70	0.005	1.47 (0.85, 2.56)	0.06	0.17	1.48 (1.06, 2.06)	0.48	0.02
MI	0.81 (0.55, 1.18)	0.53	0.27	1.78 (0.08, 39.04)	0.04	0.72	1.26 (0.54, 2.93)	0.03	0.59	0.95 (0.57, 1.58)	0.38	0.84
Stroke	1.08 (0.70, 1.67)	0.99	0.73	1.11 (0.50, 2.44)	0.90	0.8	1.46 (0.80, 2.66)	0.94	0.22	1.03 (0.54, 1.96)	0.73	0.93
All-cause mortality	0.98 (0.52, 1.83)	0.18	0.94	0.96 (0.32, 2.88)	0.23	0.94	1.39 (0.67, 2.88)	0.91	0.38	0.75 (0.46, 1.23)	0.31	0.25
Thrombosis	0.97 (0.61, 1.53)	0.06	0.88	1.60 (0.96, 2.68)	0.14	0.07	1.06 (0.74, 1.52)	0.34	0.75	0.90 (0.63, 1.28)	0.42	0.56
Bleeding	1.00 (0.88, 1.13)	0.76	0.98	0.51 (0.40, 0.66)	0.39	<0.001	1.06 (0.87, 1.28)	0.72	0.58	0.98 (0.80, 1.20)	0.55	0.83

a *P* value of Q-test for heterogeneity test.

### 2. Inclusion Criteria

The studies that met the following criteria were included: (1) published in English, (2) case- control studies on platelet activity and prospective cohort studies on clinical outcomes, (3) the evaluation of the *ABCB1* C3435T polymorphism, platelet activity and the poor clinical outcomes in patients receiving clopidogrel, (4) availability of the genotype frequency on target population, and (5) the valid date, on publication or through corresponding by e-mail, to work out an odds ratio (OR) or P-value with 95% confidence interval (CI).

**Figure 3:**
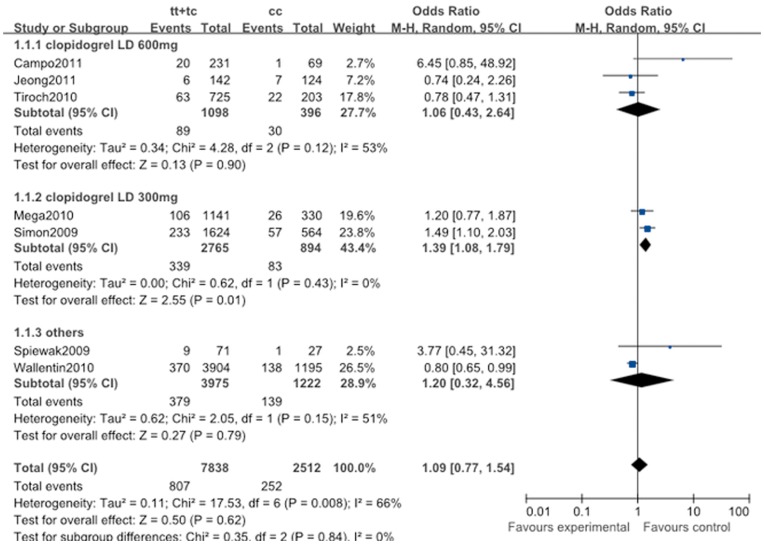
Pooled random-effects-based odds ratio of long-term major adverse cardiovascular events associated with *ABCB1* C3435T polymorphism. Comparison: TT+TC vs. CC.

### 3. Data Extraction

Two reviewers independently extracted the data and reached a consensus on all items. The following information was achieved from each study: the first author’s name, publication date, ethnicity, population studied, characteristic of target population, treatment protocal,definition of cases, poor outcomes, study period and gene information, respectively.

**Figure 4:**
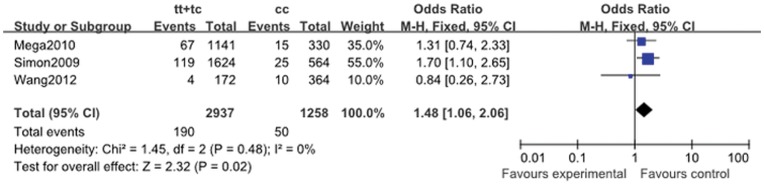
Pooled fix-effects-based odds ratio of early major adverse cardiovascular events associated with *ABCB1* C3435T polymorphism. Comparison: TT+TC vs. CC.

### 4. Study Outcomes

The two parts of endpoints (high platelet activity and poor clinical outcomes) were studied. The poor clinical outcomes included major adverse cardiovascular events (MACE) which were composed of cardiovascular death, non-fatal myocardial infarction, non-fatal stroke, as well as all-cause mortality, MI, stroke, definite or probable stent thrombosis and major or minor bleeding.

**Figure 5:**
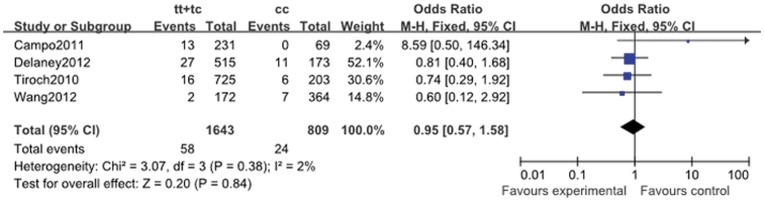
Pooled fix-effects-based odds ratio of myocardial infarction associated with *ABCB1* C3435T polymorphism. Comparison: TT+TC vs. CC.

### 5. Statistical Analysis

The observed genotype frequencies in controls or entire cohorts were tested to compare with the expected genotype frequencies by Hardy-Weinberg equilibrium (HWE). Crude odds ratio (OR) with 95% confidence interval (CI) in each study was used to assess the strength of association between *ABCB1* C3435T polymorphism and platelet activity as well as the poor clinical outcomes in patients who received clopidogrel. According to the method described by Woolf [21], the pooled ORs were assessed for allele comparison (T vs. C), dominant genetic model (CT + TT vs.CC), recessive genetic model (TT vs. CC + CT) and homozygote comparison (TT vs. CC), and its significance was evaluated by the Z-test. Heterogeneity between studies was diagnosed by the use of the χ^2^ - based Q statistic test, and regarded as significant if p value was less than 0.1 [22]. Meanwhile the statistic of I^2^ was used to efficiently test for the heterogeneity, with I^2^ less than 25%, 25–50%, and greater than 50% as low, moderate and high degree of inconsistency, respectively [23]. The fixed-effect method was adopted if the effects were appeared to be homogeneous, or the random-effect model was conducted.

**Figure 6:**
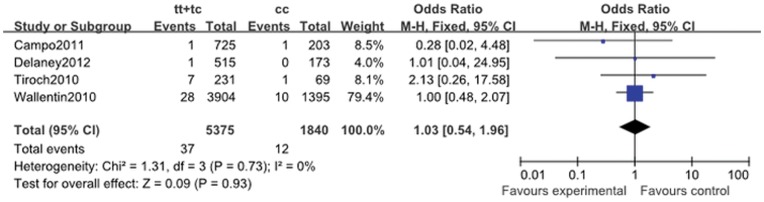
Pooled fix-effects-based odds ratio of stroke associated with *ABCB1* C3435T polymorphism. Comparison: TT+TC vs. CC.

Subgroup analyses were applied to identify the heterogeneity. Sensitivity analyses were conducted by sequential omission of individual studies respectively to detect the potential influence of each study set to the pooled ORs. In addition, publication bias was carried out by the funnel plot, and the symmetry of the plot distribution indicated the absence of publication bias [24]. Funnel-plot asymmetry was assessed with the Begg’s [25] and Egger’s [26] tests. All statistical tests were performed with the Stata (v.10.0, Stata Corporation) and Review Manager (v.5.1, The Cochrane Collaboration), and were considered significant if the 2-sided *P* value was less than 0.05.

**Figure 7:**
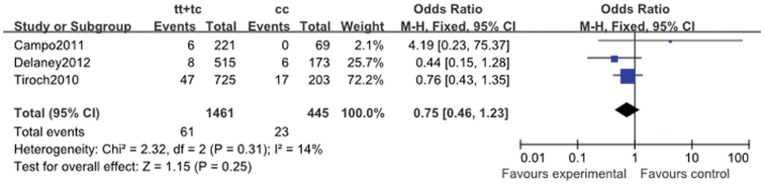
Pooled fix-effects-based odds ratio of all-cause mortality associated with *ABCB1* C3435T polymorphism. Comparison: TT+TC vs. CC.

## Results

### 1. Study Characteristics

A total of 113 studies on the *ABCB1* C3435T polymorphism with respect to platelet activity and the poor clinical outcomes were found, of which 22 replicated studies were excluded. Additionally, 39 reviews, 1 meta-analysis and 3 studies not in English were excluded. Meanwhile, 29 irrelevant studies were excluded by reviewing the title and abstract and one trial [27] was identified by screening the bibliographies. And then, five studies [28]–[32] were excluded due to their insufficient genetic information, and three trials [27], [33], [34] on clinical outcomes were excluded because they were not cohort studies. The result on platelet activity test from Wang [11] was so suspicious that we excluded it from current meta-analysis on the polymorphism and the degree of platelet inhibition by test. Finally, twelve studies, of which four involved platelet activity and ten involved clinical outcomes, met the inclusion criteria (Fig. 1), and the main characteristics of them were summarized in Tables 1, 2 and 3. Various genotyping methods were applied including allele-specific polymerase chain reaction (PCR) [10], [11], Taqman Assays[12]–[14], [16]–[20], Affymetrix Assay and Illumina Infinium Beadchip Assay [15], as well as SNPlex [9]. Distribution of genotypes in the controls or the total of each cohort (Tables 4, 5, 6, 7, 8, 9 and 10) were all not deviated from HWE.

**Figure 8:**
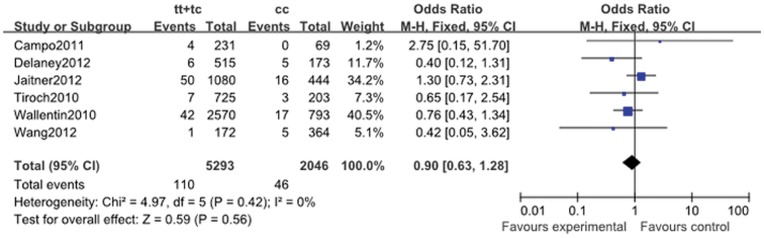
Pooled fix-effects-based odds ratio of thrombosis associated with *ABCB1* C3435T polymorphism. Comparison: TT+TC vs. CC.).

### 2. Meta-analysis Results

#### 2.1. Platelet activity

When four eligible studies were pooled, the association between platelet high activity and the *ABCB1* C3435T variation was not significant (for CT + TT vs.CC: OR, 1.01; 95% CI, 0.451 to 1.97; *P = *0.99; Fig. 2). The heterogeneity existed in allele comparison (I^2^ = 74%; *P = *0.02), homozygote comparison (I^2^ = 67%; *P* = 0.05) and dominant genetic model (I^2^ = 57%; *P* = 0.07) (Table 11).

**Figure 9:**
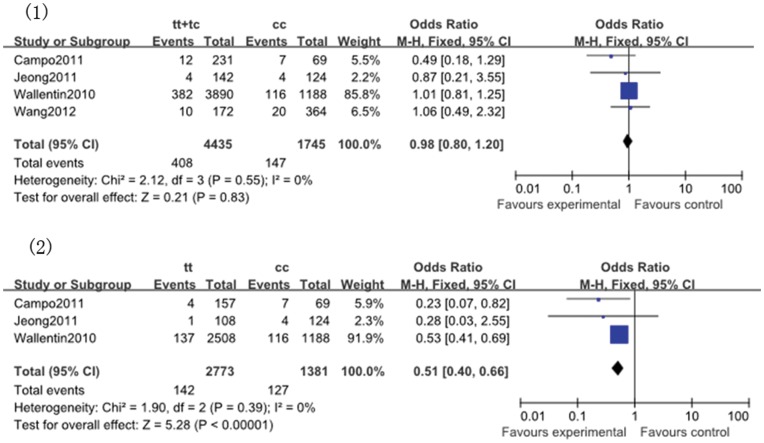
Pooled fix-effects-based odds ratio of bleeding associated with *ABCB1* C3435T polymorphism. Comparison: (1) TT+TC vs. CC;(2) TT vs. CC.

#### 2.2. Long-term major adverse cardiovascular events

The major adverse cardiovascular events (more than one year) had no significant association with *ABCB1* C3435T polymorphism in all genotype genetic models (for CT + TT vs. CC: OR, 1.09; 95% CI, 0.77 to 1.54; *P = *0.62; Fig. 3). The I^2^ statistic indicated the between-study heterogeneity (Table 11).

**Figure 10:**
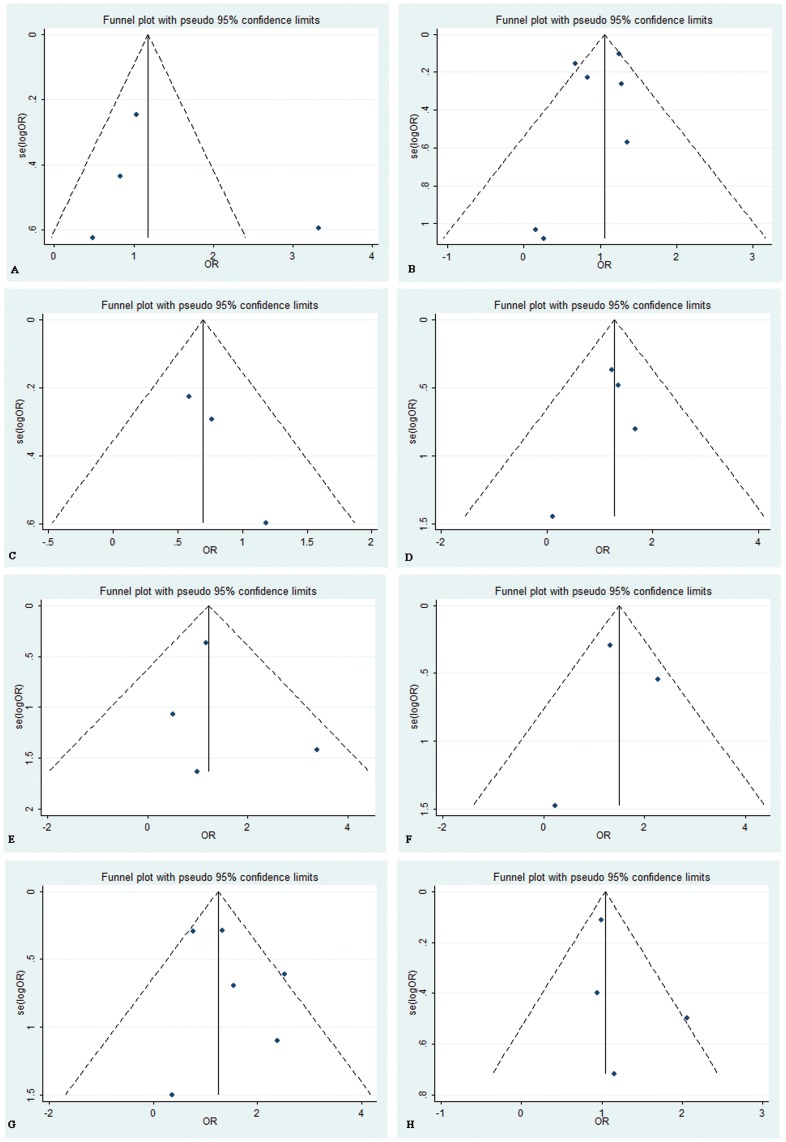
Funnel plots of the meta-analysis of *ABCB1* C3435T polymorphism (comparison: TT+TC vs. CC) and response to clopidogrel treatment. (A) platelet activity (Begg’s test, *P* = 1.000; Egger’s test, *P* = 0.402);(B) Long-term major adverse cardiovascular events (Begg’s test, *P* = 1.000; Egger’s test, *P* = 0.474);(C) Early major adverse cardiovascular events (Begg’s test, *P* = 0.296; Egger’s test, *P* = 0.124);(D) myocardial infarction (Begg’s test, *P* = 1.000; Egger’s test, *P* = 0.628);(E) stroke (Begg’s test, *P* = 0.734; Egger’s test, *P* = 0.693);(F) All-cause mortality (Begg’s test, *P* = 1.000; Egger’s test, *P* = 0.990);(G) Stent thrombosis (Begg’s test, *P = *1.000; Egger’s test, *P* = 0.372);(H) Bleeding(Begg’s test, *P* = 0.308; Egger’s test, *P = *0.425). OR = odds ratio; se = standard error.

The effect of the *ABCB1* C3435T polymorphism was further evaluated in stratification analyses. According to the clopidogrel loading dose, we found that this polymorphism was associated with the risk of the MACE in patients treated with clopidogrel LD 300 mg in allele comparison (T vs. C: OR, 1.28; 95% CI, 1.10 to 1.48; *P* = 0.001), homozygote comparison (TT vs. CC: OR, 1.59; 95% CI, 1.19 to 2.13; *P* = 0.002), and dominant genetic model (CT + TT vs. CC: OR, 1.39; 95% CI, 1.08 to 1.79; *P* = 0.01; Fig. 3). However, no significant risk of the MACE in other subgroups with this polymorphism was observed in all comparisons. Meanwhile, the heterogeneity of each subgroup was decreased (Table 11).

#### 2.3. Early major adverse cardiovascular events

Three studies provided data (MACE happened in about one month), and the heterogeneity was low for all comparisons except for that of TT vs. CC + CT (I^2^ = 72%; *P* = 0.016) (Table 11). The overall meta-analysis demonstrated that significantly elevated risk was associated with the *ABCB1* C3435T variation in allele comparison (T vs. C: OR, 1.34; 95% CI, 1.10 to 1.62; *P* = 0.003), homozygote comparison (TT vs. CC: OR, 1.77; 95% CI, 1.19 to 2.63; *P* = 0.005), and dominant genetic model (CT + TT vs.CC: OR, 1.48; 95% CI, 1.06 to 2.06; *P* = 0.02; Fig. 4).

#### 2.4. Myocadial infarction

Myocadial infarction in the five cohort studies included in the primary analysis was 5.10% (200 of the 3923 patients). The summary ORs showed no association between *ABCB1* C3435T polymorphism and risk of MI in the follow-up period (for CT + TT vs. CC: OR, 0.95; 95% CI, 0.57 to 1.58; *P* = 0.84; Fig. 5). Analysis showed that the heterogeneity existed in the homozygote comparison (I^2^ = 76%; *P* = 0.04) and recessive genetic model (I^2^ = 71%; *P* = 0.03) (Table 11).

#### 2.5. Ischemic stroke

The ischemic stroke rate in the five cohort studies was 0.69% (54 of the 7858). As described in Table 11, though no heterogeneity could be detected, the meta-analysis illustrated that *ABCB1* C3435T polymorphism was unrelated to the rate of ischemic stroke in patients treated with clopidogrel (for CT + TT vs. CC: OR, 1.03; 95% CI, 0.54 to 1.96; *P* = 0.93; Fig. 6).

#### 2.6. All-cause mortality

A total of 97 deaths (four trials, 3387 total patients) occurred during follow-up. When all eligible studies were pooled, the association between all-cause and the *ABCB1* C3435T variation was not significant (for CT + TT vs. CC: OR, 1.09; 95% CI, 0.77 to 1.54; *P* = 0.62; Fig. 7). No between-study heterogeneity was identified (Table 11).

#### 2.7. Stent thrombosis

Seven cohort studies reported stent thrombosis data (1.97%, 173 of the 8775). The cumulative incidence of stent thrombosis was not associated with *ABCB1* C3435T polymorphism in all genotype genetic models (for CT + TT vs. CC: OR, 0.90; 95% CI, 0.63 to 1.28; *P* = 0.56; Fig. 8). The heterogeneity existed in allele comparison (I^2^ = 59%; *P* = 0.06) (Table 11).

#### 2.8. Bleeding

The bleeding rate in the five cohort studies was 7.82% (596 of the 7619). The comparison of TT vs. CC was associated with a significant reduction in the outcome of bleeding (TT vs. CC: OR, 0.51; 95% CI, 0.40 to 0.66; *P*<0.00001; Fig. 9). No significance between *ABCB1* C3435T polymorphism and other genetic models as well as heterogeneity were identified (Table 11).

### 3. Test of Heterogeneity

In platelet activity studies, there was significant heterogeneity in three genetic contrasts of the *ABCB1* C3435T (Table 11). However, in the subgroup analysis, heterogeneity disappeared in studies that tested the platelet activity by light transmittance aggregometry (LTA) and the VerifyNow (T vs. C: I^2^ = 11%, *P* = 0.29; TT vs. CC: I^2^ = 0%, *P* = 0.61; TT+CT vs. CC I^2^ = 22%, *P* = 0.28).

Furthermore, significant heterogeneity existed in all the four genetic models of the *ABCB1* C3435T with long-term MACE (Table 11). But in the subgroup analysis of clopidogrel loading dose, the heterogeneity of each subgroup was changed, clopidogrel LD 300 mg in two contrasts (T vs. C: I^2^ = 0%, *P* = 0.53; TT vs. CC: I^2^ = 0%, *P* = 0.79; TT+CT vs. CC I^2^ = 0%, *P* = 0.43) except one model (TT vs. TC+CC: I^2^ = 63%, *P* = 0.10), as well as clopidogrel LD 600 mg in two contrasts (T vs. C: I^2^ = 41%, *P* = 0.19; TT vs. TC+CC: I^2^ = 19%, *P* = 0.27; TT+CT vs. CC I^2^ = 53%, *P = *0.12) except one model(TT vs. CC: I^2^ = 70%, *P* = 0.07).

In addition, the heterogeneity existed in allele comparison with regard to stent thrombosis (Table 11). When we stratified the trials by previous or current smoker percentage, the heterogeneity was not clear in the subgroup (percentage <50%) (T vs. C: I^2^ = 0%; *P* = 0.32) and the other (percentage >50%) (T vs. C: I^2^ = 34%; *P* = 0.22).

Although we also found the heterogeneity in two genetic model contrasts of Myocadial infarction and one genetic model contrast of early MACE (Table 11), due to limited studies, we failed to explain the heterogeneity. Finally, the heterogeneity could not be detected significantly in other contrasts.

### 4. Sensitivity Analysis

Sensitivity analysis was performed in the *ABCB1* C3435T dominant genetic model (CT + TT vs. CC). The significance of pooled ORs was not obviously affected by omission of individual studies except for MACE. One study [16] carried the greatest weight for long-term MACE. When it was excluded, the pooled p-values were significant in all comparisons, whereas exclusion of any other did not influence the results. Similarly, exclusion of the study by Simon *et al.* [9], the pooled OR of the dominant genetic model in early MACE was not significant. Meanwhile, in this genetic model, the heterogeneity in our meta-analysis was not influenced excessively by exclusion of any single study.

### 5. Publication Bias

Funnel plot as well as Begg’s and Egger’s tests were carried out to access the publication bias of studies. Data showed that there was no evidence of publication bias in comparison of TT+TC vs. CC (Fig. 10).

## Discussion

Clopidogrel, as a pro-drug, was known to require metabolic activation before inhibiting platelet aggregation. The *ABCB1* C3435T had been revealed to be associated with loss of function of P-glycoprotein which decreased the active metabolite of clopidogrel. On the basis of antiplatelet responding in the laboratory test and poor clinical outcomes, several molecular cardiovascular studies were conducted to evaluate the association between the *ABCB1* C3435T polymorphism and platelet response in CAD patients on clopidogrel, but the results were inconclusive. A former meta-analysis [35] showed that the association might exist between TT homozygotes of the *ABCB1* C3435T polymorphism and risk of short-term recurrent ischemic events.

In the present meta-analysis, since we included newer studies [11], [14], [19], [20] and conducted the research more meticulously with the subgroup study and more detailed trials which the former one had not included, new significance resulted. To begin with, the 3435T allele carrier was related with the risk of the early and long-tern major adverse cardiovascular events in patients treated with clopidogrel LD 300 mg. However, we did not find the significant association in subgroup clopidogrel LD 600 mg and others. Simon *et al.* [9] first found that patients with TT genotype had a higher rate of subsequent cardiovascular events than those with CC genotype. The recent clinical trial showed that compared with a 300-mg loading dose, pre-treatment with a 600-mg clopidogrel loading dose before primary PCI was associated with improvement of angiographic results and 30-day major adverse cardiovascular events [36]. The platelet response in treatment might be influenced by both *ABCB1* C3435T polymorphism and clopidogrel loading dose, which need further research to identify. Meanwhile another study [37] indicated that a 150 mg oral maintenance dose of clopidogrel resulted in more intense inhibition of platelet aggregation than a 75 mg maintenance dose, which suggested that the maintenance dose also interacted with the platelet activity. In addition, TT homozygotes decreased the outcome of bleeding compared with CC homozygotes in our meta-analysis. This was almost consistent with the result from the three respective trails [14], [16], [18]. However, one study [16] carried the greatest weight for this analyses and with limited studies included, the result should be interpreted with caution and further studies based on larger, stratified population should be examined.

Four studies [10], [12]–[14] on platelet activity tested by different methods were included in our research. This was the first meta-analysis that includes the studies on the polymorphism with the degree of platelet inhibition evaluated by empirical methods, though no significance was searched. Meanwhile, in the group of all-cause mortality, MI, Stroke, and stent thrombosis, the polymorphisms with them were also not significant. Various factors may inference these. Among them, we first pay attention to the evidence of heterogeneity, for which the reasons are unclear. It may be due to the following: the selection of methods; differences in age, gender, ethnicity, sample size; and the main clinical characteristics. For instance, the diabetes with insulin resistance lower the inhibition of platelet aggregation [38]. Various genotyping methods applied in different studies may also bring about the heterogeneity. Diverse definition of case in platelet activity tests were used, such as different time of evaluation, hence, the association mignt have been biased or simply lead to heterogeneity. If we carried out the subgroup by some of the above elements, the heterogeneity in some compares decreased.

Clopidogrel inhibits the platelet activity, and high platelet activity in patients treated with Clopidogrel indicates clopidogrel resistance or poor response to clopidogrel, which will lead to poor clinical outcome in the future. Though we have found some association between *ABCB1* C3435T polymorphism and antiplatelet responding, we also should set our insights to the interaction between single-nucleotide polymorphisms (SNP) and other factors. As coexisting, rather than single, polymorphisms in different genes may be related to persistent platelet activation while on clopidogrel [39], so gene-gene interaction, such as *P2Y12* or *CYP2C19* with *ABCB1* C3435T, should also be observed between *ABCB1* C3435T polymorphism and antiplatelet responding. On the other hand, one recent research [40] showed that the combination of a calcium channel blocker and *ABCB1 C3435T* genotype influenced the change of 20 µmol ADP-induced maximal platelet aggregation (MPA) in smoking status receiving clopidogrel. In our meta-analysis, the small scale of population and inconsistent stratification standards in environmental exposures and genotypes lowered our statistical power to further explore the gene-environment interaction. As a result, we need to give careful consideration to more sophisticated gene-gene and gene-environment interactions in a future analysis, so as to obtain a more comprehensive understanding of the association between *ABCB1* C3435T polymorphism and antiplatelet responding.

Besides, from the different aspirin doses or triple antiplatelet therapy our studies included, we believe that interaction between gene and drug combination may also exist. Prolonged use of aspirin may reduce the intestinal absorption of clopidogrel by inducing the expression of *ABCB1* in human epithelial colorectal (Caco-2) cells [41]. Researchers recently started to focus on the interactions between genetic polymorphisms and clinical effect with triple antiplatelet therapy (cilostazol, clopidogrel and aspirin) [12]. All these have urged us to pay more attention to the interaction between gene and drug combination in using clopidogrel. However, since new antiplatelet medicine had gone to market, we discovered that *ABCB1* genotypes were not significantly associated with clinical or pharmacological outcomes in patients treated with prasugrel [15] and the pharmacodynamic characteristics of ticagrelor were not influenced by *CYP2C19* and *ABCB1* genotypes [42]. These might overcome the difficulty in poor antiplatelet responding the *ABCB1* genotypes associated and gave us fresh insight to the antiplatelet treatment in addition to aspirin and clopidogrel, however numerous trails should be arranged for further assessment.

Although considerable efforts have been put into the test, there are some limitations inherent in the study. First, the number of studies included are limited, especially for the information on the risk of MACE in patients treated with clopidogrel LD 300 mg and the outcome of bleeding. Thus the conclusion about these should be considered with caution. Second, detailed information such as the ethnicity and other main characteristic are not available in some studies, which further limit our evaluation. Third, gene-gene or gene-environment interaction, different loading or maintenance dose, influence from main clinical characteristics, standardized unbiased platelet activity evaluation and genotyping methods may affect the results. These variables can be planned more effectively by a separate analysis of these elements, to which we did not have access.

In summary, our meta-analysis indicated that the *ABCB1* 3435T allele carrier was related to the risk of major adverse cardiovascular events in patients on clopidogrel LD 300 mg, and TT homozygotes decreased the outcome of bleeding compared with CC homozygotes, whereas, the association between *ABCB1* C3435T polymorphism and platelet activity as well as other risks of poor clinical outcomes were not significant. Thus, to validate our findings, additional larger studies need to focus on homogeneous cases along with standardized platelet activity evaluation and genotyping methods in further tasks.

## Supporting Information

Checklist S1 **PRISMA 2009 Checklist.**
(DOC)
